# Olfactory systems and neural circuits that modulate predator odor fear

**DOI:** 10.3389/fnbeh.2014.00072

**Published:** 2014-03-11

**Authors:** Lorey K. Takahashi

**Affiliations:** Department of Psychology, University of Hawaii at ManoaHonolulu, HI, USA

**Keywords:** predator odor, fear, main and accessory olfactory systems, amygdala, hippocampus, medial hypothalamus, medial prefrontal cortex

## Abstract

When prey animals detect the odor of a predator a constellation of fear-related autonomic, endocrine, and behavioral responses rapidly occur to facilitate survival. How olfactory sensory systems process predator odor and channel that information to specific brain circuits is a fundamental issue that is not clearly understood. However, research in the last 15 years has begun to identify some of the essential features of the sensory detection systems and brain structures that underlie predator odor fear. For instance, the main (MOS) and accessory olfactory systems (AOS) detect predator odors and different types of predator odors are sensed by specific receptors located in either the MOS or AOS. However, complex predator chemosignals may be processed by both the MOS and AOS, which complicate our understanding of the specific neural circuits connected directly and indirectly from the MOS and AOS to activate the physiological and behavioral components of unconditioned and conditioned fear. Studies indicate that brain structures including the dorsal periaqueductal gray (DPAG), paraventricular nucleus (PVN) of the hypothalamus, and the medial amygdala (MeA) appear to be broadly involved in predator odor induced autonomic activity and hypothalamic-pituitary-adrenal (HPA) stress hormone secretion. The MeA also plays a key role in predator odor unconditioned fear behavior and retrieval of contextual fear memory associated with prior predator odor experiences. Other neural structures including the bed nucleus of the stria terminalis and the ventral hippocampus (VHC) appear prominently involved in predator odor fear behavior. The basolateral amygdala (BLA), medial hypothalamic nuclei, and medial prefrontal cortex (mPFC) are also activated by some but not all predator odors. Future research that characterizes how distinct predator odors are uniquely processed in olfactory systems and neural circuits will provide significant insights into the differences of how diverse predator odors activate fear.

## Background

Fear and anxiety are activated by threat and the ability to regulate their responses is essential to adaptation and survival. Moreover, an extensive body of work indicates that abnormalities in the detection of threat may lead to pathological fear and anxiety (Lang et al., 2000; Charney, 2004; Green and Phillips, 2004; Blanchard et al., 2011; Britton et al., 2011). Thus, the biology of fear has attracted considerable attention in relation to the causal and modulatory factors linked to normal and exaggerated fear and anxiety states.

Animal models of fear that involve exposing prey to predator odor offer fundamental insights into the biology of threat detection and behavioral expression. Many species depend on olfactory sensory systems to detect a predator and engage in anti-predator behavior. Chemosensory cues or predator odors may at times be the only information available to prey that are hiding, under cover, or unable to visually detect the source of threat. Wide-ranging field and laboratory studies have discussed the diverse behavioral repertoire prey animals display when predator chemosensory cues are detected (Kats and Dill, 1998; Apfelbach et al., 2005) and the decision-making consequences prey exhibit under of risk of predation (Lima, 1998; Bytheway et al., 2013). In addition, burgeoning research is focusing on the neurobiology of predator-induced stress. In particular, exposing mice and rats to predator odor stimuli, which induces long-lasting behavioral and physiological effects, are increasingly becoming a useful, animal model that may offer insights into the pathophysiology of humans undergoing uncontrollable stress and anxiety as in post-traumatic stress disorder (Mackenzie et al., 2010; Clinchy et al., 2011; Corley et al., 2012; Matar et al., 2013).

This review highlights current research on the olfactory and neural systems that activate fear elicited by predator odors. The review begins with an overview of the olfactory systems that detect odors derived especially from predator fur, urine, and synthesized chemosignals from predators. Interconnections from olfactory systems to brain circuits activated by predator odor will then be discussed in relation to autonomic, endocrine, and fear-related unconditioned and conditioned fear.

## Diverse olfactory systems detect predator odors

The olfactory system consists of several subsystems (Breer et al., 2006; Munger et al., 2009) that include the main olfactory system (MOS), the accessory olfactory system (AOS), the septal organ of Masera, the Grueneberg ganglion (GG), and the trigeminal system. Research on predator odor detection has focused almost entirely on the two well-known MOS and AOS. A long-standing view is the MOS serves as a broadly tuned odor sensor that responds to a multitude of volatile, airborne chemicals that convey information such as the location of food and the whereabouts of predators and prey (Firestein, 2001). In contrast, the AOS mediates innate responses to non-volatile, fluid-phase chemical cues or pheromones (Meredith, 1991; Breer et al., 2006), which are chemicals released from one organism that influence the behavior or physiology of another organism of the same species (Karlson and Luscher, 1959). Pheromones likely evolved to facilitate intraspecific communication such as the reproductive and social status of a conspecific (Dulac and Torello, 2003). However, the role of the AOS has now broadened to include not only intraspecific communication but also interspecific signaling. That is, the AOS also detects the odor of non-conspecifics such as predators (Ben-Shaul et al., 2010; Papes et al., 2010) and these interspecific signaling odors are referred to as kairomones (Dicke and Grostal, 2001). Thus, both the MOS and AOS have the potential to detect predator odor and activate unconditioned fear behavior. This review section discusses our expanding knowledge of the different olfactory subsystems, especially in laboratory mice and rats, in processing predator odors.

## The main olfactory system

The MOS consists of olfactory sensory neurons in the main olfactory epithelium (MOE) that express odorant receptors (ORs) and trace amine associated receptors (TAARs) (Buck and Axel, 1991; Liberles and Buck, 2006) and project to the MOB for further olfactory information processing. The MOB then projects directly or indirectly to brain regions that modulate physiological and behavioral functions.

## The MOS of the rat and mouse appears especially sensitive in processing the predator odor trimethylthiazoline

Although only a few studies have investigated the role of the rat MOS in detecting predator odors, an important observation is the MOS does not process all types of predator odors. For example, exposure to cat odor obtained from the body or fur induces only a modest increase in Fos expression in the glomerular cell layer in the MOB (McGregor et al., 2004). In contrast, 2,4,5 dihydro 2,5 trimethylthiazoline (TMT), a highly volatile synthesize compound from red fox anal secretions induces a pronounced increase in Fos expression in both the granular layer and dorsal lateral portion of the MOB glomerular layer (Illig and Haberly, 2003; Day et al., 2004; Staples et al., 2008). An early study also reported that rats exposed to the odor of fox feces exhibited elevations in olfactory bulb mitral cell multiunit responses that were accompanied by increased vigilance, as indicated by heightened EEG and neck muscle EMG recordings and freezing behavior (Catterelli and Chanel, 1979). A role of the MOB in processing TMT is further supported in a study showing that olfactory bulb ablation effectively reduces freezing to TMT (Ayers et al., 2013).

Consistent with the limited work in rats, a number of studies in mice have further supported a role of the MOS in detecting TMT. Studies showed that intranasal perfusion of zinc sulfate, which induces transient anosmia of the MOS (McBride et al., 2003), reduces freezing and avoidance to TMT (Hacquemand et al., 2010; Galliot et al., 2012). Using genetic methods, a study reported that mutant mice with dorsal epithelium zone depletion of olfactory neurons exhibited deficits in avoidance behavior when exposed to TMT (Kobayakawa et al., 2007). Another study in mice showed that altered olfactory sensory neuron projections from the dorsal region of the olfactory epithelium to the dorsal olfactory bulb impaired avoidance behavior to TMT (Cho et al., 2011). These results suggest that a circuit in the MOS from the dorsal olfactory epithelium region to the dorsal olfactory bulb play a critical role in processing TMT.

In addition to TMT, the urine of predators is detected by the mouse MOS. One study identified an involvement of TAARs expressed in neurons of the MOE in detecting predator urine odors (Dewan et al., 2013). More specifically, this study found that genetic deletion of the olfactory TAAR gene family abolished the aversion of mice to low concentrations of volatile amines and to the odor of predator urine. Of interest, analysis of predator urine identified the volatile amine β-phenyethylamine (PEA), which is recognized by TAAR4 (Ferrero et al., 2011). Of particular relevance, mice lacking the olfactory TAAR4 exhibit deficits in their aversion to PEA (Ferrero et al., 2011; Dewan et al., 2013). These studies demonstrate predator urine contains a volatile compound, i.e., PEA, detected by a specific receptor in the MOE that induces predator fear-related behavior.

## The accessory olfactory system

The AOS consists of the vomeronasal organ (VNO), a chemoreceptive structure situated at the base of the nasal septum, which houses the microvillar vomeronasal sensory neurons (VSNs). Pheromones are detected by three classes of VSN receptors including vomeronasal receptor type 1 (V1R) and type 2 (V2R) and formyl peptide receptors (Munger et al., 2009; Liberles, 2014). The VNO sends projections to the accessory olfactory bulb (AOB), a forebrain region that serves as the first processing center of vomeronasal information. The AOB then sends information directly or indirectly to a number of brain sites involved in diverse functions.

## The rat and mouse AOS may be necessary but not sufficient in mediating predator odor processing

TMT and cat odor are prominently used to investigate the roles of the MOS and AOS in predator odor detection. As previously indicated, rats exposed to TMT, but not cat odor, showed a significant increase in Fos expression in the glomerular cell layer in the MOB. However, in striking contrast, exposure to cat odor, but not TMT, induces a robust increase in Fos expression in the glomerular, mitral, and granule cell layers of the posterior AOB (Staples et al., 2008). These results suggest that TMT is detected primarily by the MOS whereas cat odor is detected by AOS. Cat odor derived from the fur/body appears to act as a kairomone processed by the AOS.

To further assess the potentially independent role of the MOS and AOS in processing predator odor, an interesting study (Masini et al., 2010) exposed rats after destruction occurring in either the olfactory epithelium or VNO to ferret odor collected on a small towel. The study showed that rats rendered anosmic with zinc sulfate or with ablation of the VNO continued to show elevations in stress-induced corticosterone secretion when exposed to ferret odor. Only after joint destruction of the MOB and VNO did significant deficits occur in ferret odor-induced secretion of corticosterone. These results suggest the MOS and AOS have overlapping roles in modulating the ferret odor-induced increase in stress hormone secretion. Due to the reported role of the rat AOB in detecting cat odor as indicated by increased Fos expression (McGregor et al., 2004), removal of the VNO in rats would be useful to determine whether the remaining functional MOS may similarly modulate the processing of fear-related behavior induced by cat fur/body odors.

Several lines of evidence in mice also support an involvement of the VNO chemosensory system in detecting predator odor and activating fear. First, mice exposed to the urine of predators, (e.g., bobcat, fox, rat) exhibit a robust increase in electrical activity in the AOB (Ben-Shaul et al., 2010). Second, removal of the VNO or genetically impairing activation of the VNO increases the time mice spent investigating a collar worn by a cat (Samuelsen and Meredith, 2009) or the bedding of a rat (Isogai et al., 2011). Third, VN1 and VN2 receptors appear to have unique roles in detecting and identifying different types of potential predators, e.g., mammalian, reptiles, or predatory birds (Isogai et al., 2011).

The VNO role in predator odor processing was further investigated in mice lacking the TrpC2, which is the primary signal transduction channel of VNO sensory neurons. This study showed that TrpC2^−/−^ mutants exhibited deficits in avoidance and risk assessment behavior when exposed to cat odor (neck swab), snake (shed skin), and rat urine (Papes et al., 2010). Furthermore, consistent with the research implicating the olfactory dorsal epithelium in processing TMT-induced fear in mice (Kobayakawa et al., 2007), TrpC2^−/−^ mutants exposed to TMT displayed fear behavior. Thus, in the mouse and to some extent in the rat, the MOS and AOS specifically detect different types of predator odors to facilitate fear.

Of relevance in identifying specific kairomone chemosignals that activate the VNO to induce fear, investigators isolated in cat saliva the protein Feld4, a cat homolog belonging to the mouse and rat major urinary protein (MUP) family (Smith et al., 2004). The MUP family also consists of closely related proteins excreted by rodent exocrine glands (Cavaggioni and Mucignat-Caretta, 2000). Mice exposed to Feld4 exhibit an increase in both c-Fos expression in AOB and defensive behavior (Papes et al., 2010). In addition, exposure to the recombinant Feld4 ligand facilitated fear behavior and stress hormone secretion in wild type but not TrpC2^−/−^ mice with deficits in VNO signaling. The MUP ligand, cat Feld4, appears to be a chemosensory signal detected by the VNO that triggers fear in the mouse. The potential presence of Feld4 on cat fur/body during cat grooming may be the basis of the chemosensory signal that activates fear in the mouse. Generalization of the fear-eliciting effects of Feld4 from the mouse to the rat and other prey species requires further investigations.

## The mouse grueneberg ganglion detects a volatile that signals danger

In mice, the GG is a small olfactory structure located on the tip of the nose that detects odors via V2Rs, several TAARs, and ORs (Fleischer and Breer, 2010). GG neurons send axonal projections to dorsal regions of the caudal MOB (Munger et al., 2009). The mouse GG is reported to detect the intraspecific predator odor TMT and conspecific alarm pheromones or odors released when mice are killed, injured, or threatened (Brechbühl et al., 2008, 2013). Threat-induced activation of both conspecific alarm pheromones and TMT via the GG may be linked to the detection of sulfur-containing related volatiles generated by meat digestion of potential predators (Nolte et al., 1994; Brechbühl et al., 2013). These results indicate a dual role of the GG in detecting a volatile produced by both conspecifics and interspecifics that signals danger.

To determine the specific involvement of GG in activating threat, investigators found that sectioning GG axonal projections, which are sent to the MOB (Fuss et al., 2005; Koos and Fraser, 2005), reduced freezing but not risk assessment behavior when mice were exposed to volatile alarm pheromones and TMT (Brechbühl et al., 2013). The researchers suggest that although GG function is impaired, these odorants are still detected, likely by receptors in the MOE (Kobayakawa et al., 2007), which send axonal projections to MOB regions distinct from GG projections (Mamasuew et al., 2011), to maintain activation of some features of fear-related behavior.

## The potential role of the septal organ of masera in mediating predator odor has not been determined

Situated bilaterally in the septal wall between the caudal end of the VNO and the rostrally located MOE lie the septal organ of Masera (SO) (Ma, 2007). The location of the SO in the nasal cavity air path of many rodent species was hypothesized to serve as a general odor detector that alerts the individual (Giannetti et al., 1995) or plays a role in assessing food or social odors (Breer et al., 2006). Of potential interest, in mice, the SO innervates glomeruli in the olfactory bulb from the MOE (Lèvai and Strotmann, 2003), which may have relevance in detecting predator odors. An early study investigated the potential arousing or alerting function of the SO by exposing intact and SO lesioned rats to TMT and food odors as well as non-biological odors such as eucalyptol (Giannetti et al., 1995). Results indicated that both biologically relevant and meaningless odors induced similar awakening and habituation responses in intact and SO lesioned groups, which suggest the SO does not have a unique alerting functional role.

## The trigeminal system

The trigeminal system is essential in protecting against toxic or irritating odors by triggering reflexes such as apnea and sneezing. In mice and rats, noxious, pungent odors activate the trigeminal system via a large population of chemosensory cells that reach the surface of the nasal epithelium to form synaptic contacts with trigeminal afferent nerve fibers (Munger et al., 2009).

## The trigeminal system is activated by high doses of TMT but may not be involved in mediating unconditioned fear behavior

Some investigators have suggested that TMT should be classified as a general noxious odorant that activates the trigeminal system to trigger avoidance behavior (McGregor et al., 2002; Apfelbach et al., 2005; Fortes-Marco et al., 2013). Evidence suggesting a noxious, irritating role of TMT is based on work showing that exposing rats to low concentrations of TMT (35 μmol) is capable of activating the external lateral parabrachial nucleus of the trigeminal sensorial system (Day et al., 2004). In addition, 75 μmol or more of TMT stimulates stress hormone ACTH and corticosterone secretion. TMT was also reported to induce nausea in humans (Fendt et al., 2005a). Although many studies were conducted with application of large TMT doses, when low doses are used, investigators observed a pattern of murine behavior akin to unconditioned fear-related responses such as freezing and avoidance (Wallace and Rosen, 2000; Blanchard et al., 2003b; Endres et al., 2005; Hacquemand et al., 2013).

To date, only one study has specifically manipulated the trigeminal system to determine its role in mediating the effects of TMT (Ayers et al., 2013). In this study, rats with trigeminal nerve transection were exposed to TMT (300 μmol) or the noxious irritant butyric acid (900 μmol). Results indicated that although trigeminal deafferentation effectively impaired freezing to butyric acid, TMT exposed rats were spared and exhibited freezing. On the basis of these observations, TMT odors appear to activate fear via an intact MOS and not through the trigeminal system.

## Summary of the role of olfactory systems in modulating predator odor fear

In recent years, a number of investigators have begun to unravel the complex olfactory systems that process predator odors. A general conclusion of these studies is that different olfactory subsystems appear to have distinct roles in detecting different predator odors (see summary Table 1). For example, receptor systems in the MOS play a key role in mediating the sensory detection of TMT and compounds found in predator urine that activate fear. On the other hand, the AOS appears especially sensitive in processing cat odor or a potential kairomone compound found in cat saliva. However, the majority of studies that yielded insights into the specific role of olfactory subsystems were obtained almost exclusively in the mouse and broad generalization of these novel observations to other small prey species remains to be determined.

**Table 1:** **Roles of the olfactory systems in modulating predator odor fear**.

**Predator odor**	**Animal**	**Effect of odor**
**MAIN OLFACTORY SYSTEM**		
TMT	Rat	Increased Fos expression in granular layer and dorsal lateral portion of the glomerular layer (Illig and Haberly, 2003; Day et al., 2004; Staples et al., 2008).
		Olfactory bulb ablation reduced freezing (Ayers et al., 2013).
	Mouse	Transient anosmia with zinc sulfate reduced freezing and avoidance (Hacquemand et al., 2010; Galliot et al., 2012).
		Mutant mice with dorsal epithelial zone depletion of olfactory neurons exhibited deficits in avoidance behavior (Kobayakawa et al., 2007).
		Mistargeted olfactory sensory neuron projections from the dorsal region of the olfactory epithelium to the dorsal olfactory bulb showed impairments in avoidance behavior (Cho et al., 2011).
Cat fur odor	Rat	Modest increases in Fos expression in the glomerular cell layer (McGregor et al., 2004).
Predator urine	Mouse	Exposure to fox urine induced Fos expression in MOB and primary olfactory cortex (Funk and Amir, 2000).
		Genetic deletion of the TAAR gene family of main olfactory sensory neurons abolished aversion to cat urine (Dewan et al., 2013) or the amine β-phenyethylamine, identified in predator urine (Ferrero et al., 2011).
**ACCESSORY OLFACTORY SYSTEM**		
Cat fur odor	Rat	Increased Fos expression in glomerular, mitral and granule cell layers in AOB (Staples et al., 2008).
	Mouse	Ablation of VNO or genetically impairing VNO activation increased time investigating a cat collar (Samuelsen and Meredith, 2009).
Ferret fur odor	Rat	Destruction of both MOB and VNO olfactory organs is required to reduce ferret odor-induced corticosterone secretion (Masini et al., 2010).
Predator odors (cat neck swab, snake skin, rat urine)	Mouse	Mice lacking TrpC2, the signal transduction channel of VNO sensory neurons, showed deficits in avoidance and risk assessment (Papes et al., 2010).
		Exposure to cat Feld4 facilitates c-Fos expression in AOB and defensive behavior, which is blocked in TrpC2^−/−^ with impaired VNO signal processing (Papes et al., 2010).
**GRUENEBERG GANGLION**		
TMT	Mouse	Sectioning Grueneberg ganglion axonal projections to the MOB reduced freezing (Brechbühl et al., 2013).
**SEPTAL ORGAN OF MASERA**		
TMT	Mouse	No effects of septal organ lesions on alerting behavioral functions (Giannetti et al., 1995).
**TRIGEMINAL SYSTEM**		
TMT	Rat	No effects of trigeminal deafferentation on freezing (Ayers et al., 2013).

Another conclusion is that different olfactory subsystems may have complex overlapping effects in processing predator odors. Studies from the extensive sociosexual literature demonstrate that both the MOS and AOS are capable of detecting and processing both volatile and non-volatile chemosignals (Restrepo et al., 2004; Shepherd, 2006; Spehr et al., 2006; Martinez-Marcos, 2009) but differ in their signaling properties (Xu et al., 2005). Furthermore, although isolated compounds may be specifically detected by one olfactory subsystem, in natural predator odor exposure conditions complex chemosensory compounds may be present and processed by several olfactory subsystems to facilitate unconditioned fear. For example, studies showed the volatile predator urine amine PEA is detected by TAAR4 found in MOE neurons (Ferrero et al., 2011), whereas the nonvolatile kairomone MUP ligand cat Feld4 is detected by VNO neurons (Papes et al., 2010). However, the natural predator scent from the fur/body of cats and ferrets may contain a complex mixture of volatile and non-volatile sensory cues detected by the rodent MOS or AOS (see Masini et al., 2010) to induce unconditioned fear.

Similar overlapping olfactory subsystems may apply to the GG and MOS, where both olfactory subsystems express TARRs, and disruption in GG axonal projections to the MOB do not incur pronounced deficits in predator odor TMT-induced fear. Expression of V2Rs in the GG may play yet another complex role in detecting kairomone-like molecules. Thus, the complex predator body odor fur may be detected by multiple olfactory subsystems to facilitate broad activation of brain circuits that modulate unconditioned fear.

## Predator odor activates brain circuits that modulate autonomic, endocrine, and fear-related responses

This section of the review highlights some of the key brain regions linked to olfactory systems, which modulate three major components—autonomic, endocrine, and behavior—of predator odor fear or threat.

## Exposure to predator odors activate the autonomic nervous system

Exposure to threat activates the autonomic nervous system (ANS) to rapidly trigger physiological responses that facilitate immediate survival in a dangerous situation (Ulrich-Lai and Herman, 2009). Secretion of the catecholamine epinephrine and norepinephrine from the sympatho-adrenomedullary system increases heart rate, vasoconstriction, and energy mobilization to support the classic “flight or fight” response. In addition, activation of peripheral β-adrenoceptors on vagal afferents that terminate in the nucleus of the solitary tract located in the brainstem may influence locus coeruleus pathways that secrete NE throughout the brain to further support physiological, behavioral and cognitive functions (Jöels et al., 2006). Here, the few studies that examined the effects of predator odor on ANS activity are discussed (Table 2).

**Table 2:** **Effects of predator odor on autonomic and endocrine functions**.

**Predator odor**	**Animal**	**Effect of odor**
**AUTONOMIC NERVOUS SYSTEM**		
TMT	Rat	Increased sympathetic nerve activity and blood pressure (Horii et al., 2010, 2013).
Cat fur odor	Rat	Increased Fos expression in DPAG (Dielenberg and McGregor, 2001; McGregor et al., 2004).
		DPAG lesions reduced heart rate but not blood pressure (Dielenberg et al., 2004).
**ENDOCRINE SYSTEM**		
TMT	Rat	Increased HPA hormone secretion (Morrow et al., 2000; Day et al., 2004).
	Mouse	Dorsal epithelium zone depletion of olfactory neurons impaired ACTH secretion (Kobayakawa et al., 2007).
Cat fur odor	Rat	Increased HPA hormone secretion (File et al., 1993; Cohen et al., 2006; Muñoz-Abellán et al., 2011).
Ferret fur odor	Rat	Increased HPA hormone secretion (Masini et al., 2005) that is impaired after MeA lesions (Masini et al., 2009).
2-propylthietane	Rat	Increased HPA hormone secretion (Perrot-Sinal et al., 1999).

One study reported that urethane-anesthetized rats exposed to either TMT or the pungent non-predatory odor butyric acid showed a dramatic increase in adrenal sympathetic nerve activity only to TMT (Horii et al., 2010), which likely increased epinephrine section and elevated blood pressure and heart rate. The authors also showed that live rats exposed to TMT displayed an increase in freezing and a reduction in exploratory behavior. In a subsequent study (Horii et al., 2013), this research group demonstrated that rats exposed to TMT exhibited elevations not only in adrenal sympathetic nerve activity but also in body temperature, an indication of increased metabolism and another measure of elevated autonomic activity (Bouwknecht et al., 2007).

Cardiovascular functions were also studied in rats exposed to cat odor emitted from a collar worn by a cat (Dielenberg and McGregor, 2001). In this study, cat odor exposure induced a sustained increase in blood pressure but not heart rate. These autonomic responses were accompanied by a reduction in exploration, avoidance of the cat collar and heightened head out and risk-assessment activity.

Information is scarce on the role of specific neural sites underlying predator odor-induced autonomic activity. However, one brain region of interest is the dorsal periaqueductal gray (DPAG), which was implicated a number of years ago to modulate cardiovascular functions (Carobrez et al., 1983; Schenberg et al., 1983; Depaulis et al., 1992). Previous studies reported that rats exposed to cat odor exhibit an increase in Fos-positive cells in the DPAG (Dielenberg and McGregor, 2001; McGregor et al., 2004). To investigate the role of the DPAG in cat odor-induced ANS activation, rats with DPAG lesions were implanted with telemetric probes to measure heart rate and blood pressure (Dielenberg et al., 2004). When exposed to cat odor, DPAG lesioned rats showed a reduction in heart rate and locomotor activity, but no significant decrease in the cat odor-induced rise in blood pressure. The results indicate the DPAG modulates some components of the ANS activated by cat odor.

Although TMT exposure increases sympathetic nerve activity (Horii et al., 2010, 2013), the specific role of the DPAG on ANS functions are not known. Unlike cat odor showing increased Fos expression in the DPAG, exposure to TMT is not accompanied by increases in c-*fos* mRNA (Day et al., 2004), albeit recent functional magnetic resonance imaging revealed a TMT-induced increase in neural activity in the DPAG (Kessler et al., 2012). Perhaps the DPAG modulates ANS functions activated by TMT as reported with cat odor (Dielenberg et al., 2004).

## Exposure to predator odors rapidly activates the neuroendocrine stress system

In addition to facilitating of the ANS, a number of studies demonstrated that predator odor activates the hypothalamic-pituitary-adrenal system (HPA) in mice and rats (see Table 2). For example, exposure to TMT (Morrow et al., 2000; Day et al., 2004; Kobayakawa et al., 2007), cat odor (File et al., 1993; Cohen et al., 2006; Muñoz-Abellán et al., 2011), ferret odor (Masini et al., 2005) or 2-propylthietane, the main constituent of weasel anal gland secretion (Perrot-Sinal et al., 1999) increases adrenocorticotropin (ACTH) or corticosterone secretion.

The hypothalamic paraventricular nucleus (PVN) plays an important integrative role in stress by sending neuronal projections to the median eminence to regulate pituitary-adrenal hormone secretion (Sawchenko et al., 1996; Herman et al., 2002). The PVN also sends projections to brainstem sites including the parabrachial nucleus, the dorsal motor nucleus of the vagus nerve and the nucleus of the solitary tract to regulate autonomic activity (Swanson and Kuypers, 1980).

In relation to predator odor-induced HPA activation, the PVN receives information from the medial amygdala (MeA), a recipient of both direct and indirect MOS and direct AOS projections (Petrovich et al., 2001; Meredith and Westberry, 2004; Pro-Sistiaga et al., 2007). Rats with fiber-sparing lesions of the MeA exhibit significant deficits in ACTH and corticosterone secretion when exposed to ferret odor (Masini et al., 2009). However, the role of the MeA in facilitating HPA hormone secretion is not specific to predator odor. Impairment of the MeA is also reported to attenuate stress hormone secretion induced by restraint stress (Dayas et al., 1999).

Another study found that mice with dorsal epithelium zone depletion of olfactory neurons impaired ACTH secretion when exposed to TMT (Kobayakawa et al., 2007). This impairment in TMT detection and processing may disrupt a MOS-MeA-PVN circuit responsible for facilitating HPA hormone secretion. The study further indicated that dorsal epithelium zone depleted mice showed reductions in TMT-induced Zif268-positive cells in the bed nucleus of the stria terminalis (BST), anterior division, dorsal medial nucleus (BSTdm), which may reflect a disruption in a BST to PVN circuit that facilitates stress hormone secretion (Kobayakawa et al., 2007). The BST also receives direct projections from the AOB and indirect projections from the MeA (Scalia and Winans, 1975; Canteras et al., 1995; Fan and Luo, 2009) and impairments in potential odor processing in the BST may compromise activation of both HPA and ANS functions (Ulrich-Lai and Herman, 2009).

## The amygdala, especially the medial amygdala, plays an essential role in modulating predator odor unconditioned and conditioned fear

A number of reviews have discussed the neural basis of fear (e.g., LeDoux, 2000; Rosen and Donley, 2006; Pessoa and Adolphs, 2010; Gross and Canteras, 2012). A common theme of these reviews emphasizes the importance of the amygdala in threat detection, the elicitation of fear behavior, and its role in modulating fear learning and memory.

As indicated previously, the MeA receives both direct and indirect projections from olfactory systems and modulates HPA stress hormone secretion induced by predator odor. MeA cells appear sensitive to cat odor as indicated in a study showing impairments in facilitating field excitatory post synaptic potentials in the MeA of rats after exposure to cat odor (Collins, 2011). Behavioral studies demonstrate in rats that fiber-sparing lesions or temporary inactivation the MeA dramatically impair unconditioned freezing when exposed to either cat odor (Li et al., 2004; Blanchard et al., 2005) or TMT (Fendt et al., 2003). Moreover, MeA lesioned rats approached and contacted the cloth that contained cat odor and additional studies indicated MeA lesions did not produce a general increase in locomotor activity or a major deficit in olfactory detection (Li et al., 2004).

The role of the MeA was also studied in relation to predator odor contextual fear consolidation and retrieval (Takahashi et al., 2007). Rats with MeA inactivation immediately after exposure to predator odor exhibited no deficits in the consolidation of contextual fear-related behavior. However, rats with acute inactivation of the MeA immediately prior to retrieval of contextual fear showed increased approach behavior to the apparatus sector that previously contained the cat odor cloth. Thus, the MeA may have a dual role in detecting predator odor, which activates unconditioned fear, and in recalling a previous location associated with predator odor. Of note, a study showed that MeA lesioned rats are capable of freezing to an auditory stimulus paired with footshock (Nader et al., 2001) and suggests that impairment of the MeA does not produce global deficits in learned fear behavior. Rather, the MeA lesion-induced deficit in conditioned fear behavior appears specific to impairments in predator odor-context associations.

The basolateral amygdala (BLA), which is widely implicated in fear conditioning using footshock as the unconditioned stimulus paired with auditory (Fanselow and Poulos, 2005), contextual (Maren et al., 2013), or olfactory cues (Otto et al., 2000; Mouly and Sullivan, 2010), has been the focus of studies that determined whether the BLA is also involve in associating the unconditioned predator odor stimulus with the test context. Using TMT, studies in rats demonstrated that BLA inactivation or fiber-sparing lesions did not induce robust impairments in unconditioned freezing (Wallace and Rosen, 2001; Müller and Fendt, 2006) and produced only mild deficits in conditioned freezing to the context (Wallace and Rosen, 2001). However, other investigators that exposed BLA lesioned rats to cat odor reported deficits in both unconditioned freezing and approach to the cat odor (Vazdarjanova et al., 2001; Takahashi et al., 2007). In addition, temporary inactivation of the BLA immediately after exposure to cat odor impaired contextual avoidance behavior when rats were tested the next day (Takahashi et al., 2007). Thus, the BLA, which is broadly involved in emotional memory consolidation (McGaugh, 2000), is further implicated in the consolidation of cat odor-induced contextual fear.

Unlike the MeA, the BLA in rats does not receive direct projections for either the MOS or AOS (McDonald, 1998; Pitkänen, 2000). Different olfactory detection systems accompanied by downstream indirect projections to the BLA may contribute to the reported differences between TMT and cat odor in unconditioned and conditioned fear. Of possible relevance, cat and ferret fur/body odor, but not TMT, increases c-Fos expression in the BLA (Day et al., 2004; Masini et al., 2005; Staples et al., 2008), which suggests predator odor differences in activating the BLA. Nonetheless, the temporal pattern of c-Fos expression is known to vary (Redburn and Leah, 1997), and a time-course study may reveal temporal increases in c-Fos expression activated by TMT. Furthermore, although the BLA may not modulate an increase in unconditioned fear elicited by TMT, the BLA may play an alternative role in modulating the arousing effects of TMT on dorsolateral striatal-dependent response learning (Leong and Packard, 2014).

The central nucleus of the amygdala (CeA) is another target of interest in predator odor studies due to the broad role of the CeA in modulating autonomic, endocrine, and anxiety and fear behavior (Davis, 2000; LeDoux, 2000). However, studies in rats involving fiber-sparring lesions or temporary inactivation of the CeA demonstrate that exposure to either TMT (Fendt et al., 2003) or cat odor (Li et al., 2004) are ineffective in attenuating unconditioned fear behavior. Thus, not all nuclei in the amygdala are involved in modulating predator odor-induced fear behavior.

## The bed nucleus of the stria terminals modulates predator odor fear induced by olfactory information processed by the main olfactory system

Another major direct and indirect projection from olfactory systems is the BST as previously discussed. Studies revealed that inactivation of the BST, especially the ventral BST, reduces freezing when rats are exposed to TMT (Fendt et al., 2003, 2005b), or cat urine (Xu et al., 2012). Notably, the MOS plays a key role in processing both TMT (Kobayakawa et al., 2007) and predator urine (Ferrero et al., 2011; Dewan et al., 2013). Whether kairomone odors derived from predators such as from fur/body odors of the cat or ferret facilitate unconditioned fear behavior via the BST is not known, albeit exposure to both cat odor (Dielenberg and McGregor, 2001) and TMT (Day et al., 2004; Asok et al., 2013) appears to activate the BST as suggested by increased Fos expression.

## The ventral hippocampus modulates predator odor unconditioned and conditioned fear

The hippocampus plays a prominent role in emotional behavior, especially in processing contextual fear information (Kim and Fanselow, 1992; Phillips and LeDoux, 1992; Zhang et al., 2001). The ventral hippocampus (VHC) has attracted attention in odor studies due to dense reciprocal connections to the MeA and to other amygdalar nuclei such as the cortical nucleus that receives input from the MOS (Scalia and Winans, 1975; McDonald, 1998). The VHC also projects to the AOB and the piriform cortex, a major target of the MOB (Shipley and Adamek, 1984; Illig and Haberly, 2003). In rats, both the olfactory bulb and hippocampal dentate gyrus respond to weasel gland secretion 2-propylthietane and TMT by exhibiting fast wave bursts (Heale et al., 1994). TMT exposure also increases c-*fos* mRNA in the hippocampal dentate gyrus (Day et al., 2004).

Concerning predator odor unconditioned fear behavior, rats with VHC, but not dorsal (DHC), hippocampal lesions exhibited deficits in freezing and crouching when exposed to cat odor (Pentkowski et al., 2006). When tested the next day for contextual fear, VHC lesioned rats continued to show deficits in freezing. Another study in mice exposed to coyote urine showed that VHC lesions impaired avoidance and risk assessment behavior (Wang et al., 2013). In addition, VHC lesioned mice exhibited less freezing than control mice in the contextual fear test. These investigators further reported that exposure to coyote urine activates place cells in the CA1 region of the DHC and modify their firing patterns to stabilize a spatial representation of the fear eliciting encounter (Wang et al., 2012). Together, these studies in the rat and mouse using cat odor and predator urine suggest the VHC plays a role in predator odor processing of unconditioned and contextual fear and the DHC may also contribute to processing contextual fear. However, additional research is required to determine the extent to which the MOS and AOS connected to the hippocampus have overlapping or distinct roles in modulating unconditioned and conditioned predator fear behavior.

## Exposure to specific predator odors may require medial hypothalamic nuclei to activate fear behavior

Another major projection from the MeA, especially from the posteroventral MeA region, and from the BST is to medial hypothalamic nuclei, which broadly regulate reproductive, ingestive, and defensive behavior (Risold et al., 1997). Distinct medial hypothalamic nuclei consisting of the anterior hypothalamic nucleus, dorsomedial part of the ventromedial nucleus, and dorsal premammillary nucleus (PMd) are hypothesized to underlie a medial hypothalamic defensive system (Canteras, 2002).

The PMd stands out in predator studies due to robust increases in Fos expression in rats exposed to cat or cat odor (Canteras et al., 1997; Dielenberg and McGregor, 2001) and dense connections to the PAG (Cezario et al., 2008), which is involved in ANS functions and behavioral expression. Behavioral studies show that PMd lesions significantly impair cat odor-induced fear behavior (Blanchard et al., 2003a; Canteras et al., 2008). Furthermore, β-adrenoreceptor blockade in the PMd of rats prior to exposure to cat odor or prior to the context associated with cat odor effectively reduces freezing (Do Monte et al., 2008). These studies demonstrate an important role of the PMd in modulating the occurrence of predator odor unconditioned and conditioned fear.

However, some predator odors do not require the PMd to facilitate fear behavior. A study in rats showed that electrolytic lesions that damage both cells and fibers of passage through the PMd did not impair freezing to TMT (Pagani and Rosen, 2009). Furthermore, fiber-sparring lesions of the anterior and ventromedial hypothalamus failed to disrupt TMT-induced unconditioned freezing. The authors suggest the BST sends projections that pass through the anterior and ventromedial hypothalamic nuclei and circumvent the PMd to terminate in the PAG to modulate freezing expression.

This neural pathway from the BST to the PAG that circumvent connections to nuclei in the medial hypothalamus currently appears specifically activated by TMT. A study comparing the effects of cat odor and TMT on Fos expression found that exposure to cat odor, but not TMT, induced significant Fos expression in both the anterior and dorsal ventromedial hypothalamus as well and the PMd (Staples et al., 2008). This result suggests the medial hypothalamic defensive system is not broadly activated by all predator odors to modulate fear behavior.

## The medial prefrontal cortex modulates unconditioned fear elicited by predator odors

The medial prefrontal cortex (mPFC) is connected to a number of brain structures including the amygdala, hypothalamus, and periaqueductal gray (Gabbott et al., 2005; Price, 2005) that are involved in predator odor fear. In addition, the mPFC is critically involved in fear extinction (Sotres-Bayon et al., 2006; Peters et al., 2009; Marek et al., 2013).

Recent studies implicated the mPFC, consisting of the prelimbic and infralimbic cortex, in predator odor-induced unconditioned fear but the precise role of the mPFC in modulating predator odor fear is not clear. One study reported that temporary inactivation of the prelimbic region increased freezing in rats exposed to TMT (Fitzpatrick et al., 2011). However, another study showed that in 38–42 days old adolescent rats, inactivation of the prelimbic cortex impaired freezing induced by cat odor (Chan et al., 2011). Although both studies reported no significant effects of infralimbic cortex inactivation on freezing, the seemingly opposite effects of prelimbic cortex inactivation on predator odor unconditioned freezing induced by TMT and cat odor is puzzling.

In addition to the predator odor-induced behavioral differences involving the mPFC, studies indicate that exposure to cat odor activates c-Fos expression (Staples et al., 2008; Chan et al., 2011) in the mPFC, whereas no significant increases in mPFC c-Fos were found after exposure to TMT (Day et al., 2004; Staples et al., 2008; Asok et al., 2013). The mPFC of rats also showed elevations in expression of ΔFosB several days after exposure to cat odor (Mackenzie et al., 2010). In this study, expression of ΔFosB in the mPFC was associated with long-term effects of predator odor on conditioned fear. Another study measured *egr-1*, a gene transcription factor linked to learning and memory synaptic plasticity (Alberini, 2009). Of interest, rats exposed to TMT showed no significant increase in *egr-1* mRNA in the mPFC (Asok et al., 2013). Research will be required to determine how distinct predator odors such as TMT and cat odor are first processed in olfactory systems that project directly to brain structures for further processing before interacting with the mPFC to modulate unconditioned and conditioned fear behavior.

## Summary of brain circuits that modulate predator odor fear

Several distinct and overlapping brain regions have been identified that play key roles in predator odor activation of autonomic, endocrine, and fear behavior responses. For example, the MeA, which receive direct projections from MOS and AOS, appears to have a necessary role in general predator odor activation of both HPA hormone secretion and unconditioned fear behavior (see Tables 2, 3). In addition, the MeA and BLA are involved in the retrieval and consolidation, respectively, of predator odor contextual fear. The VHC, another key brain center that interacts with olfactory projection targets such as the MeA and piriform cortex, also appears to have a general role in modulating predator odor unconditioned and contextual fear behavior.

**Table 3:** **Brain structures that modulate predator odor unconditioned and conditioned fear**.

**Predator odor**	**Animal**	**Effect of odor**
**MEDIAL AMYGDALA**		
TMT	Rat	Temporary inactivation impaired unconditioned freezing (Fendt et al., 2003).
Cat fur odor	Rat	Fiber-sparring lesions impaired unconditioned freezing (Li et al., 2004; Blanchard et al., 2005).
		Temporary inactivation impaired retrieval, but not consolidation, of contextual fear (Takahashi et al., 2007).
**BASOLATERAL AMYGDALA**		
TMT	Rat	Fiber-sparring lesions do not induce robust deficits in unconditioned (Wallace and Rosen, 2001; Müller and Fendt, 2006) and conditioned freezing (Wallace and Rosen, 2001).
Cat fur odor	Rat	Lesions impaired unconditioned freezing (Vazdarjanova et al., 2001; Takahashi et al., 2007).
		Temporary inactivation immediately after training impaired contextual freezing (Takahashi et al., 2007).
**CENTRAL NUCLEUS OF AMYGDALA**		
TMT	Rat	No effects of temporary inactivation on unconditioned fear (Fendt et al., 2003).
Cat fur odor	Rat	No effects of fiber-sparring lesions on unconditioned fear (Li et al., 2004).
**BED NUCLEUS OF STRIA TERMINALIS**		
TMT	Rat	Temporary inactivation impaired unconditioned freezing (Fendt et al., 2003).
Cat urine	Rat	Temporary inactivation impaired unconditioned freezing (Xu et al., 2012).
**VENTRAL HIPPOCAMPUS**		
Cat fur odor	Rat	Lesions disrupted unconditioned and conditioned fear-related behavior (Pentkowski et al., 2006).
Coyote urine	Mouse	Lesions impaired unconditioned and conditioned fear-related behavior (Wang et al., 2013).
**MEDIAL HYPOTHALAMIC NUCLEI**		
TMT	Rat	No effects of electrolytic lesions of the dorsal premammillary nucleus on freezing (Pagani and Rosen, 2009).
		No effects of fiber-sparring lesions of anterior and ventromedial hypothalamus on freezing (Pagani and Rosen, 2009).
Cat fur odor	Rat	Dorsal premammillary nucleus lesions produce deficits in freezing (Blanchard et al., 2003a; Canteras et al., 2008).
		ß-adrenoreceptor blockade in the PMd attenuates unconditioned and conditioned contextual freezing (Do Monte et al., 2008).
**MEDIAL PREFRONTAL CORTEX**		
TMT	Rat	Inactivation of the prelimbic, but not infralimbic, cortex increases freezing (Fitzpatrick et al., 2011).
Cat fur odor	Rat	Inactivation of the prelimbic, but not infralimbic, cortex decreases freezing (Chan et al., 2011).

A number of investigators reported that exposure to cat odor induces robust contextual (Blanchard et al., 2001; Dielenberg and McGregor, 2001; Takahashi et al., 2005; Canteras et al., 2008) or auditory (Takahashi et al., 2008) fear conditioning in comparison to the less intense fear conditioning observed with TMT (Blanchard et al., 2003b; Fortes-Marco et al., 2013; Staples et al., 2008; see also ferret odor-induced conditioning in Masini et al., 2006). Notwithstanding the ability of TMT to induced contextual fear conditioning in rats tested under specific environmental conditions such as variations in test cage size (Rosen et al., 2008) or multiple pairings of TMT and the context (Endres and Fendt, 2007), behavioral testing with different predator odors are identifying some neural sites that may account for inconsistencies in displays of predator odor unconditioned and conditioned fear. For example, cat odor, but not TMT, appears to involve the BLA. Although the precise basis underlying this difference is not clear, the possibility exist that vomeronasal signal processing in the BLA may be necessary for predator odor fear conditioning. A study reported that non-volatile pheromones are especially attractive to female mice and the BLA stood out as a distinct region for vomeronal-olfactory associative learning (Moncho-Bogani et al., 2005). Furthermore, another study reported that darcin, an involatile protein sex pheromone in male mouse urine, rapidly induced conditioned preference in female mice that spent time in the site were darcin was previously detected (Roberts et al., 2012). Perhaps exposure to a predator odor kairomone with similar darcin-like properties and/or the MUP ligand, cat Feld4, will trigger rapid and robust fear conditioning in the BLA. Furthermore, predator odor fear conditioning may be enhanced by simultaneous activation of the MeA, VH, medial hypothalamic nuclei, and mPFC. That is, these nuclei are sensitive to the kairomone cat odor (see Table 3) and may require activation in conjunction with the BLA when exposed to predator odor to facilitate unconditioned and conditioned fear.

In stark contrast to the extensively studied olfactory connected neural systems in reproductive behavior or the neural systems in emotional learning and memory, based largely on work involving the application of the unconditioned footshock stimulus, knowledge on biology of predator odor fear is limited. Future predator odor research should address not only the brain circuits that modulate unconditioned and conditioned fear but also the olfactory sensory structures such as the accessory and main olfactory bulbs that are implicated in important developmental, social, and reproductive learning and memory processing (Brennan and Keverne, 1997; Landers and Sullivan, 2012). A recent study in mice reported that fear learning to threat-predictive odors involved changes in synaptic output of olfactory sensory neurons, which suggests that emotional information can be encoded at the level of primary sensory processing (Kass et al., 2013). Thus, characterizing the processing of different predator odors occurring in olfactory structures in conjunction with the complex interconnected neural circuits that mediate both the physiology and behavior of fear will be required to provide a complete picture of how predator chemosignals are uniquely processed to facilitate adaptive fear-related defensive behavior.

### Conflict of interest statement

The author declares that the research was conducted in the absence of any commercial or financial relationships that could be construed as a potential conflict of interest.
